# Supplementary material for: Gelsolin as a Potential Biomarker for Endoscopic Activity and Mucosal Healing in Ulcerative Colitis
Source: Biomedicines. 2022 Apr 9;10(4):872. doi: 10.3390/biomedicines10040872 (PMC9029534; doi:10.3390/biomedicines10040872)
Supplement: Supplementary file 1 [file biomedicines-10-00872-s001.zip › biomedicines-1662626-supplementary.pdf]

Table S1. Proteins detected in colonic samples from active UC patients.

Accession	Description	MW (kDA)	Score
P06702	Protein S100-A9	13.23351	19003.81
P30740	Leukocyte elastase inhibitor	42.7147	14394.28
P05164	Myeloperoxidase	83.81484	12962.81
P01833	Polymeric immunoglobulin receptor	83.23166	9819.803
P0DOX7	Immunoglobulin kappa light chain	23.36442	9617.495
P02788	Lactotransferrin	78.13192	9428.303
P04406	Glyceraldehyde-3-phosphate dehydrogenase	36.0304	8890.808
Q9Y6R7	IgGFc-binding protein	571.6389	8402.632
P02766	Transthyretin	15.87705	8325.644
P0CG05	Ig lambda-2 chain C regions	11.28655	8126.478
P35237	Serpin B6	42.59406	7792.275
P15086	Carboxypeptidase B	47.33778	6878.167
P05451	Lithostathine-1-alpha	18.71879	6380.899
P05109	Protein S100-A8	10.82765	6371.006
P11021	78 kDa glucose-regulated protein	72.28844	6074.402
Q02817	Mucin-2	539.9584	5919.273
P02774	Vitamin D-binding protein	52.88294	5879.984
P14618	Pyruvate kinase PKM	57.90003	5834.176
P06733	Alpha-enolase	47.13932	5613.433
P08107	Heat shock 70 kDa protein 1A/1B	70.00904	5370.469
P13796	Plastin-2	70.24389	5201.176
Q13813	Spectrin alpha chain, non-erythrocytic 1	284.3642	4764.071
P62937	Peptidyl-prolyl cis-trans isomerase A	18.00089	4705.94
P04083	Annexin A1	38.68998	4563.657
P46940	Ras GTPase-activating-like protein IQGAP1	189.1338	4414.628
A8K7I4	Calcium-activated chloride channel regulator 1	100.1631	4376.884
P00390	Glutathione reductase, mitochondrial	56.22095	4343.616
P06576	ATP synthase subunit beta, mitochondrial	56.52461	4235.408
P07237	Protein disulfide-isomerase	57.08068	4195.973
P02790	Hemopexin	51.64328	4158.718
P27797	Calreticulin	48.11183	4113.741
P04217	Alpha-1B-glycoprotein	54.21954	4025.433
P60174	Triosephosphate isomerase	30.7717	3993.095
P00441	Superoxide dismutase [Cu-Zn]	15.9259	3770.066
P06396	Gelsolin	85.64419	3749.697
P00558	Phosphoglycerate kinase 1	44.58613	3667.104
P01024	Complement C3	187.0299	3582.401
P15085	Carboxypeptidase A1	47.11078	3413.654
Q9UGM3	Deleted in malignant brain tumors 1 protein	260.5688	3310.673
P04075	Fructose-bisphosphate aldolase A	39.39531	3296.588
Q01518	Adenylyl cyclase-associated protein 1	51.8687	3272.5
P07737	Profilin-1	15.04456	3217.404
P02765	Alpha-2-HS-glycoprotein	39.31569	3090.329
P06731	Carcinoembryonic antigen-related cell adhesion molecule 5	76.74723	3079.886

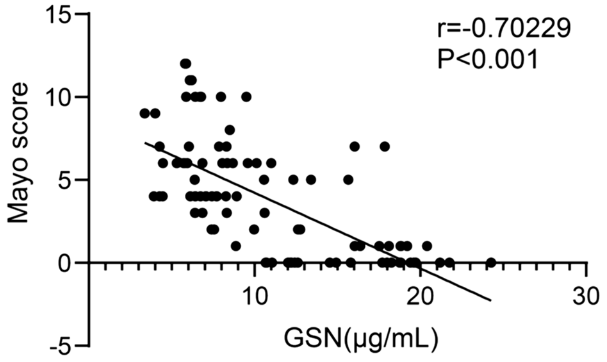

Figure S1. Correlation between the GSN level and Mayo score determined using Pearson coefficients.

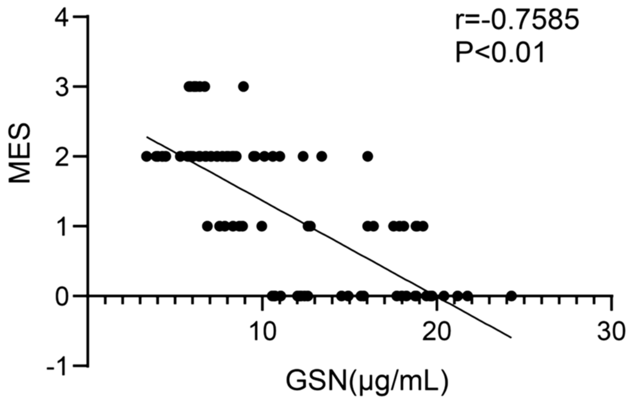

Figure S2. Correlation between the GSN level and Mayo endoscopic score determined using Pearson coefficients.

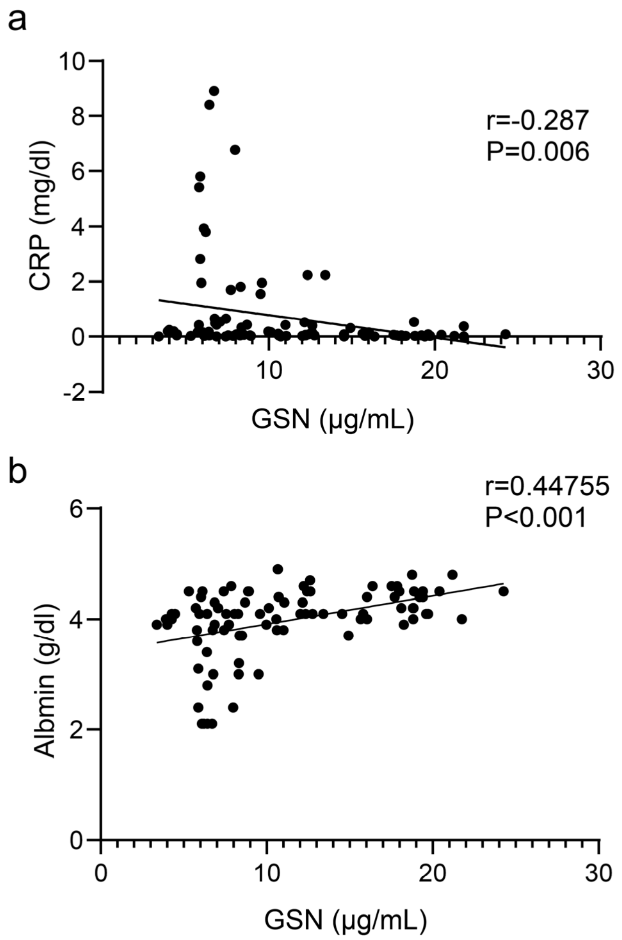

Figure S3. GSN level and C-reactive protein or albumin had a low correlation. (**a**) The correlation between the GSN level and C-reactive protein determined using Pearson coefficients. (**b**) The correlation between the GSN level and albumin determined using Pearson coefficients.
